# The Treatment of Ear-Disease by the General Practitioner

**Published:** 1894-11

**Authors:** B. Alex. Randall

**Affiliations:** Professor of Otology in the Philadelphia Polyclinic and University of Pennsylvania


### Selections and Abstracts.

---

THE TREATMENT OF EAR-DISEASE BY THE GENERAL PRACTITIONER. By B. Alex. Randall, M. A., M. D., Professor of Otology in the Philadelphia Polyclinic and University of Pennsylvania.— In a previous paper, the question of diagnosis of ear-disease was dealt with in terms as simple as could possibly give the requisite basis for rational therapeusis, and a method of record was suggested as being of great importance to the practical side of the matter. In this communication, the effort will be made to present the most concise skeleton of practical treatment, unsystematic from the text-book point of view, but resting upon the experience of the writer and aurists in general, as to what especially engages the attention of the physician and calls for unspecialized treatment.

The statistics of nearly all aural surgeons show marked accord as to the relative frequency of various affections; but such figures must be somewhat modified to truly express the findings in general practice where acute conditions are more common, as the cases are earlier seen than by the specialist. Yet for both classes of observers we may set it down as a rule that from 20-25 per cent. are affections of the external ear, nearly 70 per cent. of the middle ear and from 5-10 per cent. of the internal ear. Yet these proportions are considerably different when age and even sex are considered. In most clinics the male patients predominate in the proportion of 6 to 4

female; but the proportion is reversed when we consider the children alone—the girls being more numerous than the boys. The ratio of children to adults varies widely in different clinics—with me, they have formed 44 per cent. of my total practice. Yet such a condition as impacted wax belongs rather to adult life, being found in men as often as in women and children combined, and constituting twice as large a factor as in women and four times as large as in children, in relation to disease in general. Other affections of the external ear constitute but some 10 per cent. of the total. Acute suppuration of the middle ear, on the other hand, belongs especially to children, who constitute 63 per cent. of the cases, as well as 61 per cent. of the chronic suppurations.

What has been previously written as to the diagnosis of ear diseases, really comprises not a little of its treatment, since it will not be possible to investigate properly except through procedures which are treatment as well as exploration. Yet the precise manner of conducting most of these procedures is important enough to make, at times, all the difference between failure and success, so they may well be discussed in some detail.

The exploration of the nose and eustachian tubes calls generally for their cleansing, even when they seem little obstructed by collected secretions. This is usually best achieved with the atomizer, spraying an alkaline solution of the same specific gravity as the blood serum. If denser than this, it causes osmosis outward through the mucous membrane; if less dense, endosmosis; in each case, with decided irritation. This can best be employed through the anterior nares. A "Magic No. 2 Atomizer," costing 75 cents, serving as well as any other form, and the hand atomizer seems almost better than the more elaborate apparatus. If the tip of the nose be pressed upward by the bent thumb, while the fingers are spread upon the forehead, the tip of the atomizer will find quite a secure rest wholly external to the nose, where it can deliver its spray in any desired direction, while there is no soiling of the instrument nor a risk of injury if patient or surgeon make an unguarded movement. The bottle of the atomizer should be held between the thumb and index, the other three fingers sufficing to compress the bulb and give a practically continuous spray.

Two or three pressures will generally carry the fluid back to the pharynx, and the patient can be directed to lean forward and clear the throat by spitting, while the fluid drains from the nose. Blowing into the handkerchief should be but lightly used, and only after such removal of all fluid which might be forced up the eustachian tubes.

At times, the post-nasal syringe forms a better means of cleansing, since the warmest fluids feels cool when sprayed; and, with a tractable patient, the measure is neither very difficult nor disagreeable. Either procedure should be followed by mopping the vault of the pharynx, to complete the cleansing and generally to make an astringent medication of this most important tract. The visible lesions of the lower pharynx are generally secondary to the condition above, which especially concerns us in ear treatment. A slender cotton-carrier, bent to a right angle twenty mm. from the end, and armed with a pledget of cotton of appropriate size, should be dipped in the medicament (e. g., 2 per cent. glycerole of iodine), squeezed free of excess, and carried in as the patient makes a loud inspiration, to turn quickly up behind the velum and sweep the tube-mouths and vault clear. A tongue-depressor is generally worse than useless—in the way, rather than helpful, a great annoyance to the patient and another instrument to demand cleansing, or to risk conveying contagion. A spray of albolene medicated with menthol-camphor (2-5 per cent.), forms a good protective and mild stimulant to apply to the nares; and if inflammatory trouble is present, I have found dusting with calomel generally very efficient as a sedative, antiseptic alterative.

With the nares cleansed, we are ready for the inflation which is generally needed for exploration or treatment; but had better first study minutely the condition to be seen in the auditory meatus. Here we may find purulent matter requiring removal if furuncle of the canal or suppuration in the tympanum be present, and the use of the syringe may afford not only the best cleansing, but by the heat of the water used (105-112°) be an excellent stimulant. The pain which is often present or elicited by manipulation, yields as surely to heat thus applied as to any other means; and as this is affected by the tonic contraction of vessels and the absorption of exudate, its effect is curative as well as pallative. Drying should follow, and any clinging flakes of epidermis gently wiped away with the delicate cotton-carrier armed with a wisp of absorbent cotton. If clean, the meatus should offer no impediment to the study of the drum-head and the diagnosis of the affection and the institution of appropriate treatment. Inflation, if now practiced, should show its effect by blowing out additional secretion from the suppurating tympanum, manifesting, perhaps, a perforation previously ill-seen; or it may distend the depressed drum-head in a catarrhal case, and give relief to deafness and tinnitus previously present. In an ear apparently normal, it should serve to show normal patulency of the eustachian tube—a condition better proved than assumed.

For the inflation, the pear-shaped bag of Politzer is the appropriate instrument connected by a few inches of rubber-tubing with an olive-nozzle. With this so held as to occlude one nostril and the other closed by pressure of the fingers, the bag is pressed as the patient swallows, says "huck" or blows out the cheeks, and the air prevented from escaping from the nares, will generally force its way up to the ears. Slight pressure should be used at first, increased only if necessary.

Completion of the cleansing as perfectly as possible in the suppurating cases, with the aid of peroxide of hydrogen on the cotton pledgets and light dusting of the inflamed surfaces with impalpably powdered boric acid, completes the treatment of most of this type of cases; pneumatic massage of the drum-head in the catarrhal cases; inunction with the yellow oxide of mercury ointment in the furuncle or the eczema cases—surely this, with a few self-suggesting modifications constitutes a routine of treatment that should be within the easy execution of every practitioner. Yet this is just what forms the bulk of the work of every aurist in his office as in his clinic, and the small remainder of cases is such as tax his skill in diagnosis and treatment to the uttermost. These last can hardly be dealt with by the non-specialist.—*The Philadelphia Polyclinic.*

